# Editorial: Single-cell and spatial-omics in delineating immune-related diseases

**DOI:** 10.3389/fcell.2024.1365242

**Published:** 2024-01-16

**Authors:** Shaodi Wen, Steven Mo, Jin Zhou, Yufeng Lv, Khashayarsha Khazaie, Guangchuang Yu

**Affiliations:** ^1^ Department of Bioinformatics, School of Basic Medical Sciences, Southern Medical University, Guangzhou, China; ^2^ Department of Oncology, The Affiliated Cancer Hospital of Nanjing Medical University & Jiangsu Cancer Hospital, Nanjing, China; ^3^ Department of Basic Science, YuanDong International Academy of Life Sciences, Hong Kong, Hong Kong SAR, China; ^4^ Key Laboratory of Spatiotemporal Single-Cell Technologies and Translational Medicine, Yantai Yuhuangding Hospital of Qingdao University, Yantai, Shandong, China; ^5^ Department of Endocrinology, Yantai Yuhuangding Hospital of Qingdao University, Yantai, Shandong, China; ^6^ Department of Oncology, Foresea Life Insurance Guangxi Hospital, Nanning, China; ^7^ Departments of Immunology and Cancer Biology, Mayo Clinic, Scottsdale, AZ, United States

**Keywords:** immune-related diseases, single-cell omics, spatial transcriptome, inflamation, immune response

The immune system, a network of reciprocally interacting components, has evolved under genetic and non-genetic influences (Brodin and Davis, 2017). Nearly all pathological states in organ tissues are tied to the immune system. Infections, autoimmune diseases, immunodeficiency disorders, graft-versus-host reactions, as well as the initiation and development of cancer, all fall within the scope of immune-related diseases. One challenge in decoding the spatial structure of the disease-associated immune microenvironment is how to capture spatial molecular distribution at a high throughput approach level. Solving this problem requires the ability to record both transcriptional information and spatial coordinates. Our topic encompasses six articles focused on immune-related diseases. These papers utilize single-cell sequencing technologies and spatial multi-omics methods to investigate the intricate network relationships within immune-related diseases.

To better understand the immune response associated with Acute Respiratory Distress Syndrome (ARDS), Lu et al. highlighted that T cell subpopulation cytokines target receptors on other cells via CCL5 pathway, causing an excessive inflammatory response, which could play an important role in patients with COVID-19-induced ARDS. Moreover, the weakening of adaptive immune responses and strengthening of innate immune responses further increased the mortality rate. Similarly, infective ARDS induced by bacteria, such as bacterial/viral pneumonia ARDS and septic ARDS, remains a major clinical challenge for patients in intensive care units. Mo et al. further depicted the relationship between infection and inflammation. Specifically, ARDS caused by severe infections is more prominent among several types of immune cells with neutrophils and dendritic cells (cDCs) being higher, and macrophages (Macs) being significantly lower. The immune suppression in sepsis-induced ARDS could partially be explained by the abundance of CD8^+^ T cells, which are involved in the apoptotic signaling pathway. What’s more, in ARDS induced by acute infections, subgroups of neutrophils participate in inflammation and cytokine-related pathways, leading to their excessive infiltration into tissues, further aggravating tissue inflammation and damage in sepsis.

Non-infectious inflammation also affects the composition and function of the immune system. The single-cell transcriptome of muscle tissue and paired PBMCs from Juvenile dermatomyositis (JDM) reveal dysregulated inflammatory response in childhood systemic autoimmune conditions. Chen et al. elucidated that IFITM2^+^ and CYP4F3^+^ monocytes were largely produced in the pre-treatment group. While CYP4F3^+^ monocytes were absent in the post-treatment group. CYP4F3^+^ monocyte subclusters may represent a potential prognostic biomarker for JDM. Furthermore, CCL19^+^ fibroblasts and CD74^+^ smooth muscle cells (SMCs) were identified as inflammatory-related cell subtypes via the activation of EGR1 and/or IRF7. Particularly, intercellular communication and cell evolution during treatment provide directions and data support for basic dermatomyositis research.

The immune system plays a crucial role in transplant rejection and directly impacts the survival and functionality of transplanted organs. Wen et al. contrasted acute kidney injury (AKI) with non-rejection causes and antibody-mediated rejection (ABMR) biopsies samples. They discovered heterogeneity in the function of macrophages in renal transplant rejection responses. Specifically, macrophages can promote both damage and repair in renal allografts, depending on the severity of potential injury and the effectiveness of immunosuppressive treatment. It is recognized that inflammation is a key process leading to progressive renal fibrosis (Meng et al., 2014), however, this chronic inflammation can also lead to cardiovascular diseases, cancer, metabolic syndrome, and other diseases in transplant recipients (Nafar et al., 2011).

Inflammation and immune response are intertwined with the entire process of tumor development, including initiation, progression, treatment, drug resistance, recurrence, and metastasis (Balkwill and Mantovani, 2001; Capece et al., 2022). Approximately 80% of hepatocellular carcinoma (HCC) patients originate from viral hepatitis infection (El-Serag, 2012), further underscoring the correlation between inflammation and cancer. Research by Gao et al. suggested that the VWF gene may promote the occurrence of liver cancer associated with chronic hepatitis B and C viruses (Xiang et al., 2022) by participating in angiogenesis and negatively regulating angiogenic factors, as well as promoting angiogenesis within HCC. Additionally, HCC microenvironments harbor tumor cells with potential drug resistance. Finally, the review by Adeuyan et al. discussed single-cell and spatial multi-omics methods that could be able to identify different resistant subgroups and determine biologically significant key markers providing an important basis for early diagnosis, recurrence monitoring, therapeutic intervention, and prognosis assessment of tumors.

Multicellular organisms often have complex structures, and the spatial information between cells often determines their differentiation direction and biological functions. However, high-throughput sequencing loses that spatial information due to tissue fragmentation during the library preparation and nucleic acid extraction process, resulting in a “distortion” in the study of biological questions. The emergence of spatial transcriptomics/proteomics has added spatial coordinate information to high-throughput sequencing, partially compensating for this limitation. However, spatial omics also have some disadvantages or limitations in studying the distribution and interactions of cells within an organism, including technical limitations in sequencing depth, limited spatial resolution, subjectivity in data interpretation, and lack of direct dynamic information. Despite the rapid development of statistical methods and guidelines for sample replication, study design, and batch correction, they still lag behind. For the foreseeable future, we anticipate that spatial omics in immune-related diseases are likely to evolve across entire organisms through dynamic detection, adding more powerful and exciting tools to the immune microenvironment.

In conclusion, this Research Topic emphasizes the significance and potential advancements of single-cell and spatial multi-omics technologies in investigating immune-related diseases. It encompasses practical applications spanning various domains including infectious and non-infectious diseases, autoimmune disorders, graft-versus-host disease, and oncology. Both data manipulation techniques and biologically pertinent research within the realm of single-cell and spatial multi-omics technologies hold substantial prospects for application. We are confident that future developments will introduce innovative methodologies, explore complex mechanisms, and delve deeper into the exploration and practical implementation in clinical settings.
